# Influence of age on intestinal bile acid transport in C57BL/6 mice

**DOI:** 10.1002/prp2.287

**Published:** 2017-02-03

**Authors:** Tiandai Gao, Hirad A. Feridooni, Susan E. Howlett, Ryan M. Pelis

**Affiliations:** ^1^ Department of PharmacologyDalhousie UniversityHalifaxNova ScotiaCanada; ^2^ Department of MedicineDalhousie UniversityHalifaxNova ScotiaCanada

**Keywords:** Age, bile acid, bile acid transporters, intestine, liver, Ussing chambers

## Abstract

Intestinal and hepatic bile acid transporters are important for enterohepatic bile acid circulation and pharmacokinetics. Based on previous literature, we hypothesized that the expression of bile acid transporters and intestinal bile acid absorption are lower in older individuals. Here, we measured active taurocholate absorption across the ileum of male C57BL/6 mice in two different age cohorts – young (age range of 89–224 days) and old (age range of 613–953 days). Also examined in these mice were mRNA expression of the major bile acid transporters – Asbt and Ost*α*/*β* in the ileum, and Ntcp, Oatp1b2 and Bsep in the liver. Mean intestinal taurocholate absorption was significantly lower (~50%) in mice in the older cohort compared to those in the younger cohort. In the ileum, the expression of Asbt was significantly lower in the older cohort, but expression of Ost*α*/*β* was not affected by age. The lower capacity for intestinal bile acid absorption in the older animals is consistent with their lower expression level of Asbt. Of the hepatic bile acid transporters examined, expression of Ntcp and Oatp1b2 were significantly lower in the older mice. This is the first study to directly measure intestinal bile acid absorption as a function of age. The data suggest a lower capacity for intestinal bile acid absorption in older animals. Also, lower expression of Asbt, Ntcp, and Oatp1b2 in older individuals could influence pharmacokinetics of drug substrates.

**Abbreviation**
ASBT: apical sodium‐dependent bile acid transporter
BSEP: bile salt export pump
Cyp 7a1: cytochrome P450 7a1
FXR: farnesoid X receptor
NTCP Na^+^: taurocholate cotransporting polypeptide
OATP: organic anion transporting polypeptide
OST: organic solute transporter

## Introduction

Bile acids are important for many physiological processes, such as the emulsification of dietary fats, and the regulation of glucose, lipid, and energy metabolism (Chiang 2013). Bile acid homeostasis is maintained in part through enterohepatic circulation (Chiang 2009). Enterohepatic bile acid cycling involves the concerted activity of intestinal and hepatic bile acid transporters. Intestinal bile acid absorption is accomplished in the ileum by the apical Na^+^‐dependent bile acid transporter (ASBT) and the basolateral organic solute transporter *α*/*β* (OST*α*/*β*) (Pellicoro and Faber 2007). This is a highly efficient process, in that ~95% of the intestinal bile acid load is absorbed by these transporters (Pellicoro and Faber 2007). The Na^+^‐taurocholate cotransporting polypeptide (NTCP) and organic anion transporting polypeptides (OATPs) mediate the hepatic uptake of conjugated and unconjugated bile acids, respectively. Bile acids are transported into bile canaliculi by the ATP‐dependent bile salt export pump (BSEP) (Pellicoro and Faber 2007). Bile acid transporters are also important for the intestinal absorption and hepatic elimination of small molecule drugs, and changes in their activity, with age for example, could influence oral bioavailability (Kramer et al. 1994; Dawson et al. 2009; Han et al. 2015). Indeed, aging has a profound impact on pharmacokinetics (Mangoni and Jackson 2004).

The rationale for this study comes from a study done in young and older humans. Salemans et al. (1993) examined postprandial serum levels of bile acids in 12 older (mean of 67 years of age) and 12 younger (mean of 37 years of age) subjects. They found that the postprandial serum level of conjugated bile acids was significantly lower in the older subjects compared to the younger subjects (Salemans et al. 1993), leading them to speculate that intestinal bile acid absorption is lower in older adults. Thus, we hypothesized that intestinal bile acid absorption and the expression of bile acid transporters are lower in older individuals.

## Materials and Methods

### Chemicals and reagents

[^3^H]‐taurocholic acid with a specific activity of 10 mCi/mmol was from American Radiolabeled Chemicals, Inc. (St. Louis, MO). TaqMan gene expression assays and TaqMan Universal Master Mix II with UNG were from ThermoFisher Scientific (Burlington, ON). RNAlater, the RNeasy Mini kit, and the QuantiTect Reverse Transcription kit were from Qiagen (Toronto, ON). The Experion RNA StdSens Analysis kit was from Bio‐Rad (Mississauga, ON). All other chemicals and reagents were from Sigma‐Aldrich (Oakville, ON).

## Animals

Male C57BL/6 mice, ranging from 89 to 953 days old (~3 –31 months of age), were used in these studies. This age range in mice translates to ~20–100 years of age in human years (http://www.age-converter.com/). The mice were obtained from Charles River (St. Constant, QC, Canada). The mice were kept under a 12‐h light/dark cycle and were allowed free access to food and water. The animals were fed a standard diet (ProLab RMH 3500, LabDiet). All experiments adhered to the Canadian Council on Animal Care Guide to the Care and Use of Experimental Animals (Canadian Council on Animal Care, Ottawa, ON: Vol. 1, 2nd edition 1993:1), and all protocols were approved by the Dalhousie University Committee on Laboratory Animals.

### Tissue dissection and processing

Animals were killed by intraperitoneal injection of pentobarbital sodium 200 mg/kg with heparin 3000 U/kg. Approximately 10 min after killing, a ~1.5 cm segment of terminal ileum (proximal to the cecum) was removed. The segment of ileum was then cut into three equivalent size pieces (~0.5 cm long). The two pieces closest to the cecum were used for taurocholate transport and the piece most distal for RNA analysis. For consistency, the piece closest to the cecum was used for determination of the unidirectional lumen‐to‐blood flux (absorption) and the other for determination of the unidirectional blood‐to‐lumen flux (secretion) of taurocholate. The third piece of ileum, along with a piece of the liver (median lobe) was placed in ~20 volumes of RNAlater and stored at −80°C until processing for gene expression analysis.

### Taurocholate transport measurement

The ileum was mounted in Ussing chambers with an aperture size of 0.125 cm^2^. Each half chamber contained 1.2 mL of Kreb's buffer (containing in mmol/L: 117 NaCl, 4.5 KCl, 20 NaHCO_3_, 6 d‐glucose, 1.5 CaCl_2_, 1 MgCl_2_, 10 hydroxyethyl piperazineethanesulfonic acid, that is, HEPES; pH = 7.4). The buffer inside the chambers was maintained at 37°C and continuously stirred with magnetic stir bars. Humidified medical‐grade 95% O_2_/5% CO_2_ was continuously blown on top of the buffer to maintain pH at 7.4. Ag–AgCl electrodes were used as short‐circuiting electrodes and to measure transepithelial electrical properties of the ileum, including the transepithelial potential difference, short‐circuit current, and transepithelial resistance. The electrodes were connected to the blood and lumen side of the ileum using PE90 tubing containing solidified 3 mM KCl and 2% agar. The transepithelial potential and current were monitored using a high impedance automatic dual voltage clamp (EVC 4000‐4, World Precision Instructions, Sarasota, FL) interfaced with a Lab‐Trax‐4 and LabScribe v3 software (World Precision Instruments, Inc., Sarasota, FL) for data collection. The transepithelial resistance was determined according to Ohm's Law from the change in transepithelial potential following a brief 10 *μ*A current passed across the tissue using the voltage clamp. Transepithelial electrical properties were determined at the beginning and end of each experiment in order to monitor tissue viability.

All transport experiments were performed under voltage‐clamped conditions. A quantity of 5 *μ*mol/L taurocholate (nonradiolabeled) was added to the Kreb's solution bathing both sides of the tissue, and 0.1 *μ*mol/L ^3^H‐taurocholate was added to either the lumen or blood side of the tissue to determine the unidirectional absorptive or secretory flux, respectively. The amount of ^3^H‐taurocholate was determined by liquid scintillation spectrometry (LS6500, Beckman Coulter, CA or Tri‐Carb 2910TR, PerkinElmer, MA).

### RNA isolation, cDNA synthesis, and quantitative polymerase chain reaction

Approximately 30 mg of tissue was homogenized using a Qiagen TissueRuptor and RNA was subsequently extracted using the Qiagen RNeasy Mini Kit according to the manufacturer's instructions. RNA concentration and purity (260/280 ratio) was determined with a Cytation 3 Imaging System (BioTek, VT). RNA integrity of the samples was determined with an Experion RNA StdSens Analysis Kit and an Experion Automated Electrophoresis System (BioRad, Mississauga, ON). All samples had RNA quality indicator (RQI) values greater than 8.0. cDNA synthesis was performed with the Qiagen QuantiTect Reverse Transcription Kit. A total of 0.5 *μ*g of RNA was used in each cDNA synthesis reaction, yielding a final volume of 40 *μ*L.

Two microliters of cDNA were incubated with TaqMan Universal PCR Master Mix II and TaqMan Gene Expression Assay against a gene of interest or a housekeeping gene. qPCR was performed on a Bio‐Rad CFX Connect Real‐Time PCR Detection System using the following conditions: 50°C (2 min), 95°C (10 min), 39 cycles of 95°C (15 sec) followed by 60°C (1 min), 65°C (5 sec), and 95°C (30 sec). All C_T_ values were in the range of 15–26. The ΔΔC_T_ method was used for relative quantification of mRNA expression where mRNA expression from a 223‐day‐old mouse was set to one. In preliminary experiments, we tested the amplification efficiency of three different housekeeping genes (Eif2b1, Polr2a, and *β*2‐microglobulin) alongside the amplification efficiency of each gene of interest. Amplification efficiency plots were used to decide the appropriate housekeeping gene to use for analyzing mRNA expression of each gene of interest. *β*2‐microglobulin was found to be an appropriate housekeeping gene for all genes of interest, except Cyp7a1, which required Polr2a. All amplification efficiency plot slopes were between 0.1 and −0.1.

### Data analysis

The Shapiro–Wilk test was used to determine if mouse age, active taurocholate absorption, and relative mRNA expression levels were normally distributed. Only active taurocholate absorption was normally distributed. Thus, this influenced the statistical tests used. Since mouse age was dichotomous, the mice were separated into a young cohort and old cohort, and the effect of age on taurocholate transport was analyzed by an unpaired two‐tailed Student's *t*‐test. mRNA expression level differences between the young and old cohort were analyzed by the Mann–Whitney *U* test. All statistical analyses were performed with SPSS (version 23). Data are reported as mean ± standard error of the mean. Statistical significance was set at the *P* < 0.05 level.

## Results

**Figure 1:**
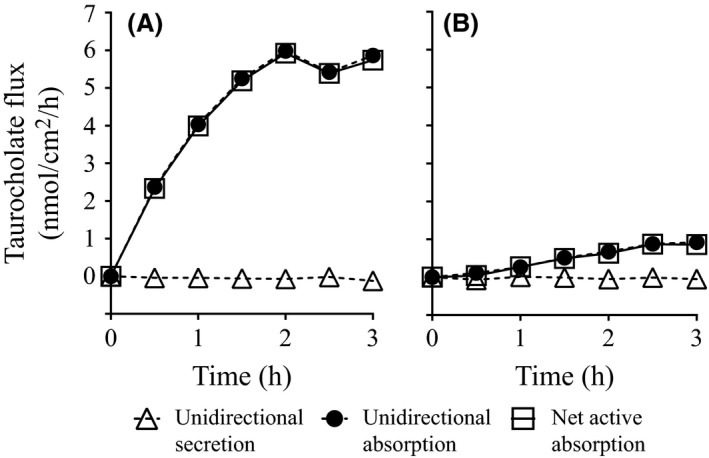
Shown are representative Ussing chamber experiments examining the unidirectional secretory flux (blood‐to‐lumen), unidirectional absorptive flux (lumen‐to‐blood), and the net active flux (the difference between the unidirectional fluxes) of taurocholate across the ileum of a young mouse (A, 209 days old) and an old mouse (B, 613 days old). In both cases, net active taurocholate transport was in the direction of absorption and reached steady state at ~2 h. The steady‐state active taurocholate absorption rate was greater in the young mouse compared to the old mouse. Experiments were conducted under voltage‐clamped conditions with 5 *μ*mol/L taurocholate bathing both sides of the tissue.

To examine the functional activity of bile acid transporters (Asbt and Ost*α*/*β*) in the ileum, we measured taurocholate flux across the ileum of mice of varying ages. Figure 1 shows representative experiments of transepithelial taurocholate transport by the ileum from a young mouse (209 days old, Fig. 1A) compared to an old mouse (613 days old, Fig. 1B). In both cases, the unidirectional secretory flux (blood‐to‐lumen flux) was low. In contrast, the unidirectional absorptive flux (lumen‐to‐blood) was large. In both cases, net active transepithelial taurocholate transport, which is the difference between the unidirectional fluxes, was in the direction of absorption and reached steady‐state at ~2 h. The ileum from the young animal showed a greater capacity for active taurocholate absorption compared to the ileum from the older animal.

**Figure 2:**
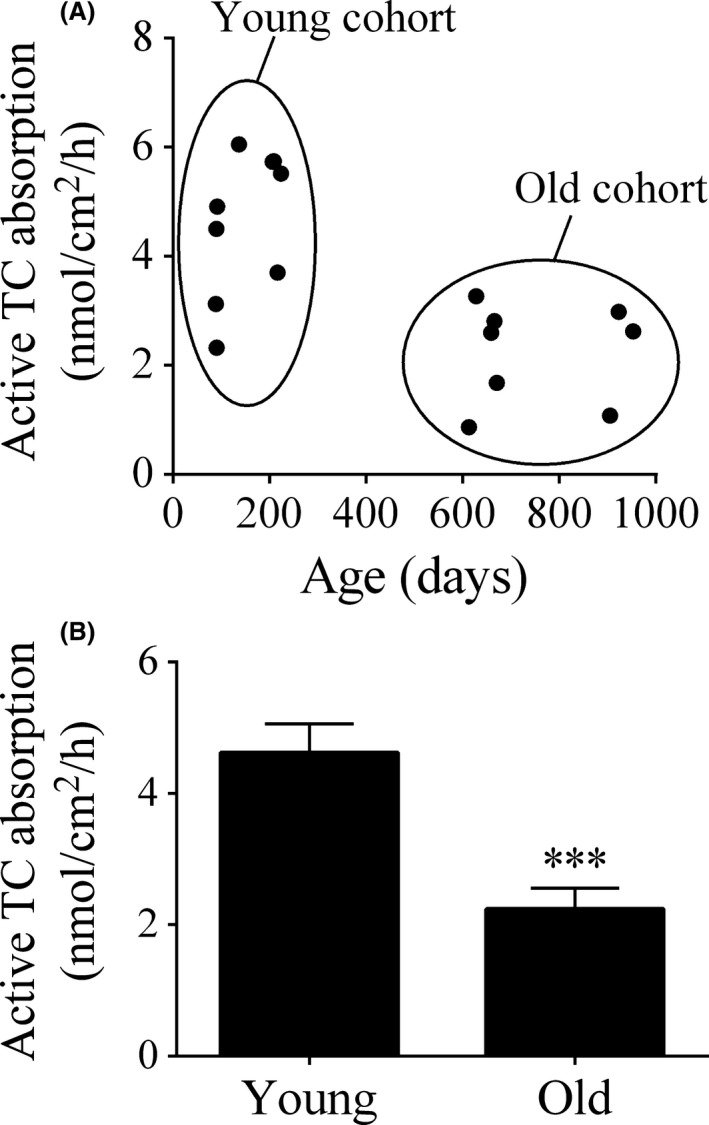
Shown is active taurocholate absorption at steady‐state by the ileum in Ussing chambers as a function of age. (A) Individual data points for active taurocholate (TC) absorption by the ileum from the mice in the young and old cohorts. (B) Mean active taurocholate absorption by the ileum of young versus old mice. Ileum from mice in the young cohort (age range of 89–224 days, *n* = 9) had significantly greater active taurocholate absorption than mice in the old cohort (age range of 613–953 days, *n* = 8), *P *<* *0.001, two‐tailed unpaired Student's *t*‐test. ***, *P *<* *0.001

Similar time courses of unidirectional taurocholate fluxes across the ileum were performed for all of the mice in our study group, and the net active absorption at steady‐state (2–3 h) was used for subsequent analysis. Figure 2A shows active taurocholate absorption by the ileum from each of the animals examined. The difference between the highest and lowest active taurocholate absorption rate was ~7‐fold. Since mouse age was dichotomous, we separated the mice into two groups – a young cohort (age range of 89–224 days, average of 174 days, *n* = 9) and an old cohort (age range of 613–953 days, average of 752 days, *n* = 8). The mean active taurocholate absorption rate across the mouse ileum was ~50% significantly lower in the old cohort compared to the young cohort (*P* < 0.001, two‐tailed unpaired Student's *t*‐test, Fig. 2B). There was no statistical difference in the tissue transepithelial potential difference (1.9 ± 0.27 vs. 1.4 ± 0.19 mV, lumen positive), transepithelial resistance (71.4 ± 6.4 vs. 56.1 ± 4.3 Ω cm^2^), or short‐circuit current (−14.9 ± 2.8 vs. −9.1 ± 1.5 *μ*A/cm^2^) between the young and old cohort (*P *>* *0.05, two‐tailed unpaired Student's *t*‐test).

**Figure 3:**
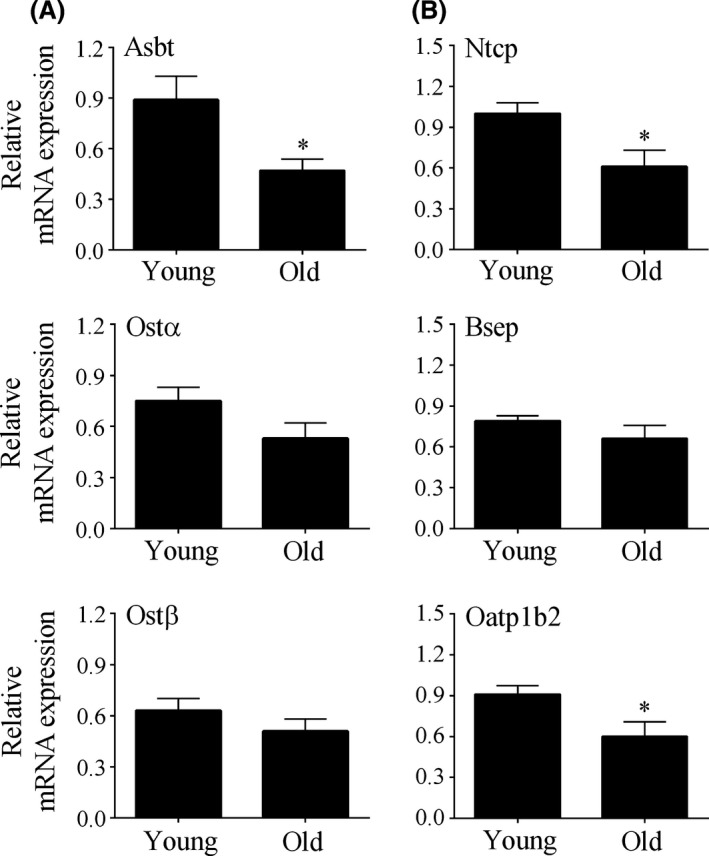
Relative mRNA expression of intestinal (Asbt, Ost*α*, and Ost*β*, Panel A) and hepatic (Ntcp, Bsep and Oatp1b2, Panel B) bile acid transporters in the young cohort compared to the old cohort. The mRNA expression of the genes was expressed relative to the expression level of a 223‐day old mouse, which was set to 1. In the ileum, there was a significant difference in the expression of Asbt (*P *<* *0.05, Mann–Whitney *U* test), but not in the expression of Ost*α* or Ost*β*. In the liver, there was a significant difference in the expression of Ntcp and Oatp1b2 (*P *<* *0.05, Mann–Whitney U test), but not in Bsep. *, *P *<* *0.05.

We also examined the mRNA expression of bile acid transporters in the ileum and liver of mice in the two cohorts. Consistent with the lower rate of taurocholate absorption, there was a ~50% lower level of Asbt mRNA in the ileum from mice in the old cohort (Fig. 3A). However, the mRNA expression of Ost*α* and Ost*β* was not different between the two cohorts (Fig. 3A). The mRNA expression of the hepatic bile acid uptake transporters, Ntcp and Oatp1b2, were significantly lower in the liver of old mice, but there was no effect of age on the mRNA expression of Bsep in the liver (Fig. 3B). Although not shown, the mRNA expression of Fxr in the intestine, and the mRNA expression of Fxr and Cyp7a1 in the liver were not affected by age (data not shown). Fxr is a bile acid nuclear receptor that regulates the expression of proteins involved in enterohepatic bile acid circulation, and Cyp7a1 is the rate‐limiting enzyme in bile acid synthesis (Chiang 2013).

## Discussion

There is indirect evidence in the literature for reduced intestinal bile acid absorption in older adult humans compared to young individuals (Salemans et al. 1993). However, no study to date has directly measured intestinal bile acid absorption as a function of age. Here, we measured ileal bile acid absorption in young and old C57BL/6 mice, and also examined the mRNA expression level of the most pertinent bile acid transporters. We found that active taurocholate absorption was lower in older mice. Consistent with these data, the mRNA expression of the apical bile acid uptake transporter, Asbt, was lower in older mice, whereas the expression of the basolateral efflux transporter, Ost*α*/*β*, was not influenced by age. Given that bile acid transport by Asbt is the rate‐limiting step in intestinal bile acid absorption (Hofmann et al. 1995), its lower expression level in older animals provides a mechanism for the lower rate of intestinal bile acid absorption. Interestingly, serum levels of unconjugated and conjugated bile acids were found to be relatively constant with aging in male C57BL/6 mice (Fu et al. 2012).

A study by Fu et al. (2012) also examined Asbt and Ost*α*/*β* mRNA expression in the ileum of male C57BL/6 mice ranging in age from 3 to 27 months. Although the data were not shown, they noted that expression of Asbt and Ost*α*/*β* were relatively stable with age. A possible explanation for differences in our results and theirs is that they took the posterior one‐third of the small intestine for expression analysis, whereas we took a ~0.5 cm segment near the cecum and adjacent to the segment that was used for taurocholate transport measurement. Importantly, Asbt expression in the mouse gastrointestinal tract is almost entirely restricted to the ileum (Cheng and Klaassen 2009), but within the ileum, there could be regional differences in its expression, which may have contributed to a discrepancy in results.

In addition to Asbt, the mRNA expression of the hepatic bile acid uptake transporters, Ntcp and Oatp1b2, were lower in older animals, whereas we did not see an effect of age on Bsep expression. Two other studies have examined the mRNA expression of Ntcp, Oatp1b2, and Bsep in livers of male C57BL/6 mice as a function of age (Fu et al. 2012)(Zhang et al. 2010). Similar to our results, Zhang et al. (2010) observed a lower expression of Ntcp and Oatp1b2 mRNA in old mice (24 months of age) compared to young mice (12 months of age). In contrast, the expression of Ntcp and Oatp1b2 were relatively constant from 3 to 27 months of age in the study by Fu et al. (2012). With respect to Bsep, studies have either found no effect of age (this study and Fu et al. 2012) or a lower expression in older compared to younger animals (Zhang et al. 2010). The reasons for discrepancies between studies are unclear.

In addition to their physiological importance, bile acid transporters are also potential drug targets, and influence pharmacokinetics as well. ASBT is a potential target for increasing the intestinal absorption of prodrugs carrying a bile acid moiety (Tolle‐Sander et al. 2004). ASBT inhibitors that reduce intestinal bile acid absorption have a blood glucose‐lowering effect, and could be developed as a novel treatment for Type II diabetes (Chen et al. 2012). Reduced bile acid absorption in older individuals would be expected to have a blood glucose lowering effect, and may partly explain why hypoglycemia is common in older people with diabetes (Abdelhafiz et al. 2015).

NTCP has a relatively broad substrate selectivity, interacting with numerous drugs, including select statins, and therefore likely plays a role in their hepatic uptake and elimination (Anwer and Stieger 2014). OATPs in the liver are important uptake transporters involved in elimination from the body of numerous drugs, including statins, sartanes, antibiotics, and anticancer drugs (Stieger and Hagenbuch 2014). Importantly, a decrease in the hepatic expression of NTCP and OATPs in older adult humans could lead to reduced hepatic elimination and an increase in body exposure to drug substrates, such as statins. Indeed, adverse reactions to drugs such as statins are a common occurrence in older adult humans (Marcum et al. 2012).

In summary, we showed that intestinal bile acid absorption is lower in older compared to younger animals. The decrease in bile acid absorption is likely the result of decreased expression of the rate‐limiting intestinal bile acid uptake transporter, Asbt. Also, the hepatic expression of Ntcp and Oatp1b2 was lower in the older mice. Changes in the expression and function of ileal and hepatic bile acid transporters with age likely have important physiological and pharmacokinetic implications.

## Authorship Contributions

Participated in research design: Gao, Howlett, and Pelis. Conducted experiments: Gao, Feridooni, and Pelis. Performed data analysis: Gao, Feridooni, and Pelis. Wrote or contributed to the writing of the manuscript: Gao, Howlett, Ferridooni and Pelis.

## Conflict of Interest

None declared.
